# Value of endometrial biopsy in patients with hysteroscopically atrophic endometrium in patients with postmenopausal bleeding

**DOI:** 10.1007/s00404-024-07922-3

**Published:** 2025-01-20

**Authors:** Ayisha A. Ashmore, Abayomi I. Alao, Amie Hibbard, Libbi Burchnall, Natalie Menic, Summi Abdul, Viren Asher, Anish Bali, Shilpa Kolhe, Andrew Phillips

**Affiliations:** 1 Derby Gynaecological Cancer Centre, Department of Gynaecology, University Hospitals of Derby and Burton NHS Foundation Trust (UHDB), Royal Derby Hospital, Uttoxeter Road, Derby, DE22 3NE UK; 2 University of Nottingham, Nottingham, UK

**Keywords:** Endometrial cancer, Atrophic endometrium, Hysteroscopy

## Abstract

**Purpose:**

To determine the rate of precancer and cancer in women presenting with PMB who have a visually atrophic endometrium at hysteroscopy and assess the value of endometrial biopsy in this situation and the adequacy of the samples obtained.

**Methods:**

Retrospective reviews of all patients with a visually atrophic endometrium at hysteroscopy who had presented with PMB and had an ET > / = 4 mm or ET < 4 mm with focal changes or irregular features between 2013 and 2024 at University Hospitals of Derby and Burton were included (*n* = 1096). Patients who had previously had cancer or precancer or had unclear hysteroscopy findings were excluded. The endometrial biopsy histology result was considered the main outcome measure.

**Results:**

188 patients did not have a biopsy performed (17.15%), 660 patients had benign pathology (60.22%), and 239 patients had an inadequate sample result (21.81%). Nine patients had precancerous changes (0.82%). The rate of cancer was 0.00% (*n* = 0). The NPV of a visually atrophic endometrial cavity at hysteroscopy in detecting precancer or cancer was 99.2%. Patients with an ET < 4 mm pre-hysteroscopy and an atrophic endometrial cavity at hysteroscopy were 2.25 times more likely than those whose ET is > 4 mm to have an inadequate sample (*p* < 0.001, 95% CI 1.61–3.16). 10 patients who had an inadequate sample at initial biopsy had a repeat inadequate sample (*n* = 23, 43.48%).

**Conclusions:**

The incidence of precancer/cancer in patients presenting with PMB with a visually atrophic endometrium at hysteroscopy is low. Many patients within this cohort have an inadequate sample at biopsy, and therefore, repeat sampling is of questionable value.

## What does this study add to the clinical work

**Table:** 

There were no instances of cancer in patients with an hysteroscopically atrophic endometrium. This study provides evidence to support clinicians in not performing a biopsy within the context of a hysteroscopically atrophic endometrium as all other evidence pertains to ultrasound findings.	

## Introduction

Post-menopausal bleeding (PMB) is the most common presentation of endometrial cancer [1]. In the United Kingdom (UK), women with PMB are initially referred for a transvaginal ultrasound to assess the thickness of endometrium. Those with an endometrial thickness (ET) of < 4 mm with an absence of irregular features are unlikely to have cancer or precancer and can therefore be reassured [1]. In women with an ET of > / = 4 mm or containing irregular features, an endometrial biopsy is required for further investigation of potential malignant change [1]. Most patients are offered an outpatient Pipelle® biopsy, but for many, this may not be feasible or inappropriate given irregular ultrasound features or significant risk factors for cancer [1]. In such cases, hysteroscopy is recommended to visualise the endometrial cavity and obtain a biopsy [1].

The accuracy of hysteroscopy in diagnosing endometrial cancer and hyperplasia has been reported to be variable. In a systematic review of data in 26,346 women, a positive hysteroscopy result increased the probability of cancer by 67.9% but a negative result only reduced the probability of cancer by 3.3% [2].

Therefore, a biopsy is particularly important in those with a negative hysteroscopy result to exclude malignant changes. However, obtaining an adequate endometrial biopsy relies on operator experience and can be challenging when the endometrial thickness is low [3]. This difficulty is further compounded when the endometrial cavity is atrophic on visual inspection and many samples are reported as inadequate. There is no consensus on the value of an endometrial biopsy in the context of a clinically atrophic uterus, and indeed, many samples taken in this context are inadequate. Further, when a sample is reported as inadequate, there is uncertainty as to whether a repeat biopsy is required.

## Aim/theory

This study aims to determine the rate of precancerous changes such as endometrial hyperplasia and cancer in women presenting with PMB who have a visually atrophic endometrium at hysteroscopy and assess the value of endometrial biopsy in this situation and the adequacy of the samples obtained.

## Method

The study was registered and approved as a retrospective study with the Department of Obstetrics and Gynaecology at University Hospitals of Derby and Burton (UHDB) in accordance with the hospital research and development guidance. All patient data were obtained with permission from the UHDB Caldicott Guardian, ensuring patient confidentiality and compliance with data protection regulations. Patients were identified from the prospectively recorded hospital outpatient hysteroscopy databases.

We performed a retrospective review of all patients who had been referred for hysteroscopy due to postmenopausal bleeding and were reported to have an atrophic endometrial cavity between 2013 and 2023. This included both outpatient and inpatient hysteroscopy patients. All histological specimens were assessed by specialist gynaecological histopathologists.

All women were offered treatment in line with the UK guidance and followed up in accordance with accepted national and local guideline algorithms [1, 4]. Inclusion criteria were: women with PMB with an ET > / = 4 mm or ET < 4 mm with focal changes or irregular features. Exclusion criteria were: previous cancer or precancerous changes under histological follow-up and poor views at hysteroscopy resulting in inability to make an adequate assessment.

Statistical analyses were performed using SPSS 26. Incidence of precancer and cancer were the primary outcome variables. χ^2^ was performed to test for differences between histology outcome and both operator grade and type of biopsy performed. Binomial logistic regression was performed to determine whether there was an association between pre-hysteroscopy endometrial thickness and incidence of an inadequate biopsy sample in patients with a visually atrophic endometrium on hysteroscopy.

## Results

A total of 1096 patients were identified between 2013 and 2023. The median age of patients was 57 years and the mean BMI was 29.5 kg/m^2^. The median ET in all patients was 5.0 mm.

### Histology

One hundred and eighty-eight patients did not have a biopsy performed due to clinical findings of an atrophic uterus (17.1%). 660 patients had a benign pathology (60.22%) on histological examination of their biopsy, but 239 patients had an inadequate sample result (21.81%). Of those that had a biopsy, two patients had endometrial hyperplasia (EH) with atypia and seven patients had EH without atypia. The rate of cancer within the dataset was 0.00% (*n* = 0). The rate of precancer was 0.82% (*n* = 9). See Table 1. Of those that did not have a biopsy or had an inadequate sample, none went on to have cancer or precancer. The negative predictive value of a visually atrophic endometrial cavity at hysteroscopy in detecting precancer or cancer was 99.01%.

**Table 1:** Histology results from endometrial biopsy

Histology	Total (*n*)	Rate (%)
Atrophic or inactive endometrium	349	31.84
Benign polyp	98	8.95
Chronic inflammation/endometritis	12	1.09
EH with atypia	2	0.18
EH without atypia	7	0.64
Secretory endometrium (progesterone effect)	20	1.82
Proliferative endometrium	143	13.05
Other benign	37	3.38
Inadequate sample	239	21.81
No biopsy performed	189	17.24
Grand total	1096	100.00

### Risk factors

Precancer rate was sub-analyzed by risk factors including Body Mass Index (BMI), Hormone Replacement Therapy (HRT), diabetes, nulliparity, and use of tamoxifen (see Table 2). The highest rate of precancer was seen in the nulliparous cohort with 2.20% of patients having precancer (*n* = 2). In patients with BMI of over 30 kg/m^2^, the precancer rate was 1.35%. The precancer rate in BMI class 1 was 0.07% (*n* = 1, total 135), BMI class 2 was 0.00% (*n* = 0, total = 76), and BMI class 3 was 3.53% (*n* = 3, total 85). In those with a normal-overweight BMI category, the precancer rate was 0.63% (*n* = 5, total = 800).

**Table 2:** Precancer rate by risk factor

Risk factor	Total (*n*)	Total precancer (n)	Rate of precancer (%)
BMI > 30	296	4	1.35
HRT	104	0	0.00
Diabetes	55	0	0.00
Nulliparity	91	2	2.20
Tamoxifen	39	2	5.13

### Complex features on ultrasound

544 patients had complex features on ultrasound scan including a heterogeneous endometrium, intracavity fluid, an irregular endometrial–myometrial junction, or a suspected focal lesion, see Table 3.

**Table 3:** Precancer rate by ultrasound feature

Type of complex feature	Total (*n*)	Total precancer (*n*)	Rate of precancer (%)
Heterogeneous endometrium	179	1	0.56
Intracavity fluid	98	0	0.00
Irregular endometrial–myometrial junction	35	0	0.00
Suspected focal lesion	232	3	1.29
No complex features	552	5	0.91
Grand total	1096	9	0.82

### Inadequate samples

907 patients (82.76%) of patient had a biopsy performed. 239 patients (21.8%) were reported to have had an inadequate sample on histological examination.

In patients who had a biopsy performed, 756 patients (68.98%) had a Pipelle® biopsy only, 63 patients (5.75%) had a directed biopsy only, and 62 patients (5.66%) had both a Pipelle® and directed biopsy. 16 patients (1.46%) had endometrial curettings (Table 4). The rest of the patients did not have a biopsy, or their mode of biopsy was unknown (18.15%). In patients who had a Pipelle® biopsy, 28.4% (*n* = 215) had an inadequate sample result. In patients who had a directed biopsy, 15.87% (*n* = 10) had an inadequate sample result. χ^2^ testing found a statistically significant difference in the number of inadequate histology results returned between Pipelle® sampling and direct sampling modalities (*p* = 0.03).

**Table 4:** Type of biopsy

Type of sample	Type of biopsy (n)					
	Directed	Pipelle®	Directed and Pipelle®	Curettings	Unknown	Total
Adequate sample	53	541	54	12	8	668
Inadequate sample	10	215	8	4	2	239
Grand total	63	756	62	16	10	907

772 patients out of 907 patients (85.12%) had a biopsy performed by either a consultant or advanced nurse practitioner. 135 patients (14.88%) had biopsies performed by a registrar or the operator grade was unknown. χ^2^ testing did not find a statistically significant difference in the number of inadequate histology results returned between operator grades of advanced nurse practitioner or consultant (*p* = 0.98).

Twenty-three patients who had an inadequate sample at initial biopsy had a repeat biopsy performed. Ten of these samples were again reported as inadequate (43.48%). The remaining repeat samples showed benign pathology only.

When the pre-hysteroscopy ET was < 4 mm (but had focal changes or irregular features), the rate of inadequate samples was higher at 26.99% (*n* = 78, total ET < 4 mm = 289). A binomial logistic regression was performed to ascertain the association between a pre-hysteroscopy ET of > / = 4 mm and the likelihood that patients have an inadequate sample. The logistic regression model was statistically significant in our population of patients who were found to have an atrophic endometrial cavity on hysteroscopy χ2(df 1) = 21.41, *p* < 0.001. The model explained 3.7% (Nagelkerke R^2^) of the variance in inadequate samples and correctly classified 74.2% of cases. The model predicted the odds of an inadequate sample in patients with an ET < 4 mm pre-hysteroscopy and found to have an atrophic endometrial cavity at hysteroscopy is 2.25 times more than those whose ET is > 4 mm. This was statistically significant (*p* < 0.001, 95% CI 1.61–3.16).

## Discussion

### Main findings

We present the largest study of patients undergoing hysteroscopy with clinically atrophic findings. In this context, patients and clinicians can be assured that the rate of cancer can be effectively excluded on hysteroscopic findings alone and the rate of precancer in these patients is extremely low. In our dataset, there were no occurrences of endometrial cancer in patients who presented with PMB and had an atrophic endometrium of hysteroscopy. We also found that the incidence of precancer was very low in this cohort of patients (0.82%, *n* = 9).

One of the problems with performing biopsies in patients with atrophic endometrial cavities at hysteroscopy is that success is dependent on operator experience. Often samples will be reported as ‘inadequate’, and it is therefore challenging to plan further management. Our study found that there was a higher proportion of patients with an inadequate sample when a Pipelle® biopsy technique was used in comparison with a directed biopsy technique using 5Fr hysteroscopic grasper (*p* < 0.05). However, there was no significant difference between number of inadequate samples when the operator was a consultant or an advanced nurse practitioner (*p* > 0.05). Of those that had an initial inadequate sample result, almost half had a repeat inadequate sample. No instances of precancer or cancer were found in repeat biopsy samples. We also found that patients with a pre-hysteroscopy ET of < 4 mm were 2.25 times more likely to have an inadequate sample at biopsy than those with an ET > / = 4 mm when the hysteroscopic finding was of an atrophic endometrial cavity. As such, we suggest that in the atrophic endometrium with satisfactory view, biopsies are of limited value and certainly a repeat biopsy due to inadequate pathology is of no value.

### Strengths and limitations

Our study has the largest cohort of patients with PMB and a visually atrophic endometrial cavity at hysteroscopy. However, it suffers from the usual limitations of a retrospective single center review which makes it susceptible to bias. We therefore propose a larger study in the context of a randomised control trial to fully assess the value of endometrial biopsy in the context of an atrophic endometrial cavity at hysteroscopy with no other abnormal endometrial features on ultrasound.

### Interpretation

There is no UK guidance on management of patients with an inadequate biopsy sample at hysteroscopy. Our study, however, suggests that an inadequate biopsy in the context of a clinical atrophic uterus is reassuring to the patient, and as such, they should be discharged without repeat sampling. Indeed, this is supported by a meta-analysis evidencing high sensitivity of hysteroscopic impression in diagnosing EC (82.6%, specificity 99.7%) [5]. However, in the context of recurrent PMB, this situation is more challenging with the British Gynaecological Cancer Society recommending hysteroscopy and endometrial biopsy in patients where there is recurrent PMB and by extension also recommending a hysterectomy in patients where there is unexplained recurrent PMB [1]. While our data support the safety of finding an atrophic endometrium at hysteroscopy, in the context of recurrent unexplained bleeding further studies are required to investigate the value of such findings.

One UK study of 97 patients presenting with PMB found that successfully obtaining a Pipelle® sample could avoid the need for a hysteroscopy altogether in 61.5% of cases [3]. They reported no cases of missed endometrial cancer when a Pipelle® biopsy was successfully obtained. However, the authors also reported that success of Pipelle® biopsy is dependent on the ET. The study found the probability of obtaining an adequate sample in patients with an ET of < 5 mm was only 27%. The authors therefore recommended that an endometrial biopsy should only be performed when the ET is > 4 mm as there is little value and success in garnering a sample when the ET is less than this.

Similarly, a study of 166 postmenopausal patients in Italy found that patients with an ET < 7 mm were more likely to have an atrophic endometrium at histology [6]. The authors, therefore, concluded that hysteroscopy and biopsy diagnostic testing should be used with caution and consideration.

However, a prospective study of 913 Dutch women presenting with PMB had alternative conclusions [7]. The authors found that 66 out of 913 patients had insufficient tissue obtained from office endometrial biopsy. Of these women, repeat sampling revealed 3 endometrial cancer and 1 endometrial hyperplasia. The study concluded that a non-diagnostic endometrial sample does not exclude cancer, and thus, further endometrial sampling is advised.

Endometrial biopsy technique can also be considered when trying to obtain an adequate sample. There is evidence that similar problems with obtaining adequate samples exist with the other forms of blind pipette-type biopsies. One study reported an inadequate rate of 27% with the MedGyn® pipette [8]. Direct sampling techniques seem to have more success and a further study found a more accurate diagnosis of EC histology using a hysteroscopic guided ‘grasp’ biopsy compared with a blind Novak curette [9]. These findings are supported by a recent review of evidence of recommendations which have demonstrated the superiority of directed biopsy techniques such as ‘chip’ or ‘pick-up’ biopsies in women with atrophic endometrial cavities in obtaining an adequate sample [10, 11]. Interestingly, when comparing final histological diagnosis following surgery to the initial endometrial biopsy, both volume of tissue and accuracy of sampling seem to be important. A meta-analysis of 12,459 patients showed that there was higher agreement between hysteroscopic biopsy and final diagnosis compared with dilatation and curettage despite dilatation and curettage producing a higher volume of tissue (*p* = 0.02) [12]. Essentially, a larger biopsy and directed biopsies are much more likely to be adequate and generate an accurate histological diagnosis [13].

Patients’ expectations and perceptions of care are equally important when there is uncertainty surrounding management. Hysteroscopies and/or biopsies can be uncomfortable and outpatient procedures in particular have been associated with significant pain, anxiety, and embarrassment [14–17]. However, there are also reports that women with PMB are strongly risk averse and would prefer to have investigations to avoid missing an endometrial cancer, even if the risk of missing this diagnosis was low (0.4%) [18]. Thus, we suggest that if a biopsy is being considered in the context of an atrophic endometrium, a directed biopsy is taken instead of a blind Pipelle® biopsy.

Our findings have significant resource implications for the NHS. The cost per endometrial biopsy is £98 based on NHS tariffs [2, 19]. In our cohort alone, the cost of all biopsies performed was £88,886 where the vast majority of samples were benign. Further, £23,442 could have been saved from potentially unnecessary biopsies that were reported as inadequate. Additionally, there are wider implications on cancer waiting list times and faster diagnostics standards. Processing time for any biopsy will extend the time that patients remain on a cancer pathway which may not confer any benefit according to the results of our study.

Our study highlighted that the highest rate of precancer in patients with a hysteroscopically atrophic endometrium was in those taking tamoxifen (*n* = 2 out of 39, 5.13%). Although these numbers are small, they represent a high-risk group and careful consideration regarding the need for biopsy should be given to this cohort of patients. In fact, in patients taking tamoxifen, there is a plausible risk of EC in both pre-menopausal and postmenopausal women who may have a thickened endometrium with or without PMB [20, 21]. The risk of EC can be predicted using various parameters of which hysteroscopic findings are chief among them, and so, further investigation is required to assess whether our results are generalisable [21].

Finally, we must stress that our results are only applicable to those patients which meet our inclusion criteria. In our initial data collection, two cancers were identified. On further examination of the patients’ records, the first patient was under routine follow-up for atypical endometrial hyperplasia and the second had poor views of the cavity due to blood and no ostia were seen. These patients were thereby excluded from our study.

In conclusion, our study found no evidence of endometrial cancer in patients with a visually atrophic endometrial cavity on hysteroscopy. A directed endometrial biopsy using hysteroscopic 5Fr grasper is considered superior to blind Pipelle® biopsy to obtain histological confirmation of benign endometrium. We also found that one-fifth of patients had an inadequate sample at biopsy and almost half of repeat samples were also inadequate. We believe that given the lack of evidence governing the management of patients with inadequate samples, further evidence is required to support repeat invasive diagnostic testing. We believe that currently our results support a more conservative approach, but any change in policy must be guided by high-quality randomized evidence.

## Data Availability

Anonymised data can be provided on request.
